# A Global Health Immersion Program for Nursing and Public Health Students

**DOI:** 10.5334/aogh.4746

**Published:** 2025-07-18

**Authors:** Taryn Vian, Sebaka Malope, Elizabeth Nkabane-Nkholongo, Jill E. Sanders, Brian W. Jack, Laura Chyu

**Affiliations:** 1 University of San Francisco, San Francisco, CA, USA; 2 Lone Mountain Global, LLP, MA, USA; 3 Lesotho-Boston Health Alliance, Maseru, Kingdom of Lesotho; 4 Boston University School of Medicine Boston, MA, USA; 5 Sefako Makgatho University, Department of Public Health, Pretoria, Republic of South Africa

**Keywords:** global health education, curriculum, global nursing, international cooperation, learning, service, cultural immersion, Lesotho

## Abstract

*Background:* The University of San Francisco, California (USFCA) and the Lesotho-Boston Health Alliance (LeBoHA) collaborated in designing and implementing an intensive, interprofessional global health immersion program for nursing and public health students from the United States. The program focused on health systems strengthening in Lesotho. This article reports on the curriculum development process, learning outcomes, and lessons learned from implementing the program.

*Methods:* USFCA and LeBoHA began collaborating in March 2023. The partners codeveloped curriculum with the goal of introducing US students to global health systems challenges and ways to address them in a resource-constrained setting. The program sought to facilitate interprofessional collaboration between undergraduate nursing and graduate public health students, promote cross-cultural awareness and humility, and foster global perspectives and relationships to create a healthier and more humane world. The activity prioritized mutual respect in how the partner institutions worked together.

*Results:* Three nursing and five public health students participated in the program held in January 2024 in Hlotse, Leribe District, Lesotho. The program included classroom learning sessions, health facility site visits, and extracurricular activities. Evaluation data suggested that students achieved course objectives and appreciated the active, experiential learning format. Lessons learned include planning for additional field visits and expanding the experience, possibly through Sesotho language instruction and interaction between students and LeBoHA faculty/staff before arrival and after the program ends.

*Conclusion:* Short-term global health immersions can help to prepare nursing and public health students for careers in global health or working with underserved communities in the United States and strengthen interprofessionalism. Immersion programs should be developed in partnership and provide reciprocal benefits to the institutions involved.

## Introduction

Global learning is defined as “the process of diverse students working together to analyze and address complex issues that transcend borders of all kinds [1].” In the United States, short-term experiences in global health are increasingly popular among medical students [2–4], though less than 10% of US undergraduate students study abroad [5]. Yet, students in the health professions at undergraduate and graduate levels can benefit from opportunities for short-term international experiences. For example, dental students participated in an oral health immersion in Ethiopia [6], and nursing students gained competencies in interprofessionalism and cultural humility during global immersions in Tanzania and Belize [7–9].

Global immersion programs challenge students to view complex problems from multiple perspectives and build communication skills [10]. Students deepen their understanding of how resource constraints and social determinants of health affect population health outcomes [3]. Immersions also help build students’ confidence in and commitment to serving the needs of culturally diverse populations and clarify their own career goals [3, 4, 11, 12]. Short-term (under 30 days) global programs often share the goal of preparing globally engaged health-care professionals from higher-income countries to understand the challenges faced in resource-constrained settings and work more effectively with underserved populations [2, 11, 13].

Given global health’s focus on equity and caring and cooperative societal relationships, the design of educational programs should follow principles of respect and a commitment to mutual benefits [14, 15]. Best practices include “bidirectional partnership,” that is, the institution sending students should work collaboratively with the partner institution on program design and implementation decisions so that both institutions accrue benefits [4]. The partner institution receives fair financial reimbursement for their involvement with the program administration complying with local cultural and other operating norms [2, 16]. Programs conducting research or providing clinical services must obtain local operating approvals and informed consent from participants and patients [6]. Key curricular components are study, respectful engagement, and an opportunity for students to reflect [3, 6, 13].

In March 2023, the University of San Francisco, California (USFCA) began collaborating with the Lesotho-Boston Health Alliance (LeBoHA), to design a short-term global health experience in Lesotho for USFCA students. The USFCA School of Nursing and Health Professions ranks in the top 3% of undergraduate nursing programs in the United States and is first in ethnic diversity among US national universities [17]. In the Jesuit Catholic tradition, USFCA seeks to promote the common good by critically, thoughtfully, and innovatively addressing inequities to create a more humane and just world. LeBoHA is a nonprofit organization (public trust), established in 2007, and registered in 2020 as an institute of higher learning, with the mission to strengthen management and clinical capacity in the Lesotho health sector by strengthening health professions education and professional development with a focus on district hospitals and primary health care. Building on strong relationships with the Lesotho Ministry of Health and Boston University School of Medicine, LeBoHA endeavors include postgraduate education in family medicine [18], a national medical internship program [19], continuing education for nurses [20], and building capacity for problem-solving and quality improvement at all levels of the health-care system [21].

## Methods

### Study design

This study is a qualitative case study of an intensive, interprofessional global health immersion program. Qualitative inquiry is appropriate as we seek to understand patterns, themes, and meaning from the perspectives of people involved in or knowledgeable about the topic [22]. In work settings, qualitative methods are applied to questions on organizational processes, including program implementation. Organizational case studies are valued for shedding light on the details of social processes in context [23]. Qualitative case studies can help generate theories or hypotheses about how concepts interrelate [24].

USFCA immersion programs seek to foster global perspectives and relationships so that students can “fully integrate and apply their knowledge, values, and skills in the pursuit of personal and professional fulfillment, and the creation of a more humane and just world [25].” The credit-bearing immersion program in Lesotho was designed for undergraduate or graduate students from any discipline interested in the challenges facing health systems in a resource-constrained country. The Kingdom of Lesotho contends with health challenges, including HIV/AIDS, tuberculosis, malnutrition, infant and maternal mortality, and shortages of health workers [26]. In partnership with LeBoHA, the global health immersion would help students understand the history and progress of health system strengthening efforts in Lesotho, and experience how a local institution is working to improve health and reduce disparities in access to care and health outcomes.

USFCA originally proposed a program focused on quality improvement training. However, during design discussions, LeBoHA leaders questioned the feasibility of this topic, given the need to partner with Lesotho health professionals, collect and analyze data, and make team-based decisions within a quick time limit. LeBoHA proposed to focus instead on the components of the health system and LeBoHA’ s current and past work in strengthening health systems.

### Ethical considerations

The study is considered exempt research due to the educational setting and the fact that it involves normal educational practices that are not likely to adversely impact students’ opportunities to learn [27]. The instruments include standard tools to study the effectiveness of instructional techniques, curricula, or classroom management methods.

### Objectives

The program sought to increase students’ skills and competencies in five areas:

1. Describe the major causes of morbidity and mortality in Lesotho;
2. Identify challenges and possible solutions for health programs in resource-limited settings;
3. Critically analyze the role of non profit organizations, government, and international development partners in health systems strengthening;
4. Practice values of cultural humility and cultural competency in an international setting;
5. Reflect on the knowledge, skills, abilities, and characteristics needed to serve or work professionally in global health.

### Program description

**Figure 1:**
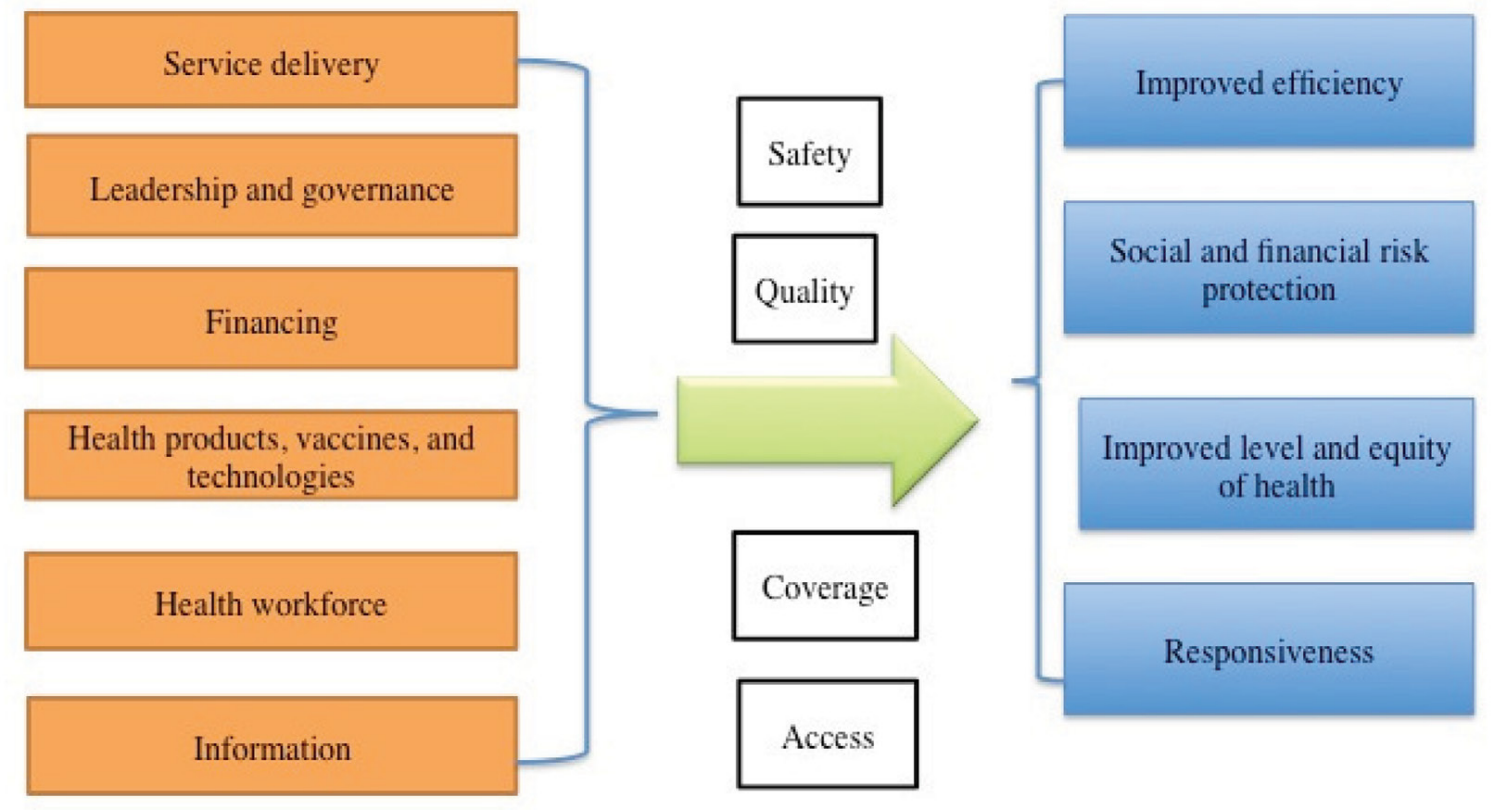
World Health Organization Health Systems Building Blocks Framework. *Source*: World Health Organization (WHO). Everybody’s business—strengthening health systems to improve health outcomes: WHO’s framework for action. WHO. 2007.

The two-week program was designed for up to 12 students. Successful completion earned 2–4 elective credits toward undergraduate or graduate degree programs and provided 50 h of applied practice experience for Master of Public Health students. The program was not designed to provide clinical services. It was designed around the WHO Health Systems Building Blocks Framework (Figure 1) and encouraged students to apply systems thinking to achieve health goals.

The budget was developed collaboratively by USFCA and LeBoHA to provide adequate reimbursement for local costs, including food, accommodation, transportation, teaching equipment, and faculty/staff time. USFCA was responsible for the salary and travel expenses for USFCA faculty and staff, and for student books and supplies. USFCA oversaw student recruitment. Each student paid tuition, a program fee (calculated to cover local expenses), and airfare. Students were eligible for a $1,000 scholarship award, which all students in the program applied for and received.

Learning sessions were held in the LeBoHA Academic Center near Motebang Hospital, a regional hospital in the peri-urban district of Leribe, Lesotho. The Academic Center was newly built with funding from United States Agency for International Development (USAID) American Schools and Hospitals Abroad program.

USFCA provided several hours of orientation to students before departure, including in-person and online orientations on Lesotho and LeBoHA, program objectives, schedule, and assignments; safety and health considerations; cultural awareness and appropriate dress; and other topics to help students prepare. A trained study abroad staff advisor attended the orientation and joined the program participants in Lesotho.

## Results

Three undergraduate nursing and five graduate public health students participated in the program held on January 2–16, 2024, in Hlotse, Leribe District. The group included seven women and one man, with the average age of 26 years (range: 19–36 years). The nursing students had completed at least 1.5 years of undergraduate study; two of the five public health students had completed one semester (8 credits), while the other public health students had completed four semesters (~30 credits).

The program included instructional sessions, site visits to two hospitals and a health center, and a visit to a center for disabled children and adjacent primary school. Extracurricular activities included a visit to Katse Dam, hikes, and a social event. See Table 1 for the full schedule and the intended purpose of the activities. One faculty member from USFCA; the Executive Director of LeBoHA; and guest lecturers from LeBoHA, Motebang Hospital, and the Leribe District Health Management Team led didactic sessions on Lesotho’s burden of disease, health systems components, and LeBoHA’s programs and activities. USFCA and LeBoHA faculty and staff attended the site visits and weekend field trips and interacted socially with students over meals and other activities, creating friendly relationships.

**Table 1:** Lesotho Global Health Immersion Schedule of Activities.

DATE	ACTIVITIES	PURPOSE OF ACTIVITY
**Tue., Jan. 2**	Arrival and tour of LeBoHA HQ in Maseru	Help students become familiar with the local partner and capital city of Lesotho
**Wed., Jan. 3**	Tour of Queen Mamohato Memorial Hospital (national hospital, formerly a public–private partnership)	Introduce the types of staffing and services provided in the highest-level hospital in Lesotho, compared with US hospitals
**Thur., Jan. 4**	Travel to Leribe (~2-h drive)	See the terrain of Lesotho; view roads and how transportation can be a barrier to access to health services
**Fri., Jan. 5**	Course overview; burden of disease in Lesotho Motebang Hospital tour	Identify measures of population health, data sources, and how health issues differ in Lesotho compared to the United States (e.g., HIV/AIDS, TB, and maternal health) View the layout, staffing, services, and patient flow of a regional hospital
**Sat., Jan. 6**	Visit to Katse Dam	Observe rural and remote areas of Lesotho, including roads and housing Gain appreciation of governance issues in access to safe water and costs and benefits of alternative water supplies
**Sun., Jan. 7**	Study and free time	Time for observation and reflection
**Mon., Jan. 8**	**Overview of health systems** Session Review (student assignment) WHO Health System Framework, LeBoHA history Teach back (student assignment)	Learn about the WHO health system building blocks, and LeBoHA projects and its relationship with government; experience problems and problem-solving (delays and electrical outages) and teatime and meals with LeBoHA staff
**Tue., Jan. 9**	**Community health and quality improvement** Session Review (student assignment) Pontmain Health Center visit, Phelisanong Centre for Disabled Children visit Quality improvement and nursing programs Teach back (student assignment)	Experience the staffing, layout, services, and quality issues at a rural health clinic; view and discuss facilities for supporting disabled children; meet nursing staff and appreciate the different levels of training and practice in Lesotho versus the United States for nursing and public health
**Wed., Jan. 10**	**Health professions education and training** Session Review (student assignment) Internship and FMSTP programs Center for Excellence in PHC Teach back (student assignment)	Appreciate how LeBoHA works with the government in organizing and provided health professions education at different levels, including reform challenges and successes
**Thu., Jan. 11**	**Service delivery and district health management** Session Review (student assignment) District health management Behavioral health in Lesotho Teach back (student assignment)	Meet a District Health Officer and appreciate the role and responsibilities of the District Health Management Team in overseeing district services, including mental health; discuss differences between community health services in the United States and Lesotho
**Fri., Jan. 12**	**Monitoring, evaluation, and financial systems** Session Review (student assignment) Monitoring, evaluation, and financing Students conduct research for final projects Teach back (student assignment)	Meet the monitoring and finance officers; discuss problems in calculating burden of disease; discuss Lesotho’s health financing system in relation to the goals of Universal Health Care; work with local faculty to choose a topic for the final project and begin research
**Sat., Jan. 13**	Tshehlanyane National Park	Interact informally with staff and members of the community; appreciate the customs, environment, and natural beauty of Lesotho
**Sun., Jan. 14**	Work on final project presentations	Apply and integrate health systems knowledge to describe a real problem affecting health services in Lesotho; research causes and viable solutions from other countries or other NGOs locally; develop recommendations
**Mon., Jan. 15**	Student presentations Feedback on course Hike Dinner and dancing	Apply skills in oral communication, visual presentation, and leading discussions Reflect on course learning Spend time with LeBoHA staff during a strenuous hike and a celebration with music, dancing, and food
**Tue., Jan. 16**	Departure	Reflect on the immersion experience, lessons learned, and re-integration

*Notes*: HQ: headquarters; LeBoHA: Lesotho-Boston Health Alliance; HC: health center; FMSTP: Family Medicine Specialty Training Program; PHC: primary health care.

### Assessment of learning

Student learning was promoted and assessed through five assignments. See Supplementary file for full descriptions.

***Book Review.*** Students wrote answers to questions about “*Everything Lost is Found Again: Four Seasons in Lesotho*” by Will McGrath, a series of essays on life and culture in Lesotho. Students described how the book portrayed the hardships and inequalities experienced by the people of Lesotho as well as community strengths and assets. Several stories touched on families affected by HIV/AIDS, introducing students to the challenges that this disease has created for Lesotho and its people. Quotes below show how students wrote about local customs and social norms.

- *[In one story,] the adults were dancing, talking, singing, and laughing at the kindergarten graduation. The adults placed so much value on this because living past five years old is a huge milestone… [McGrath wrote that] people in the Western world “feel entitled to... long lives” but Basotho people do not [take this for granted].*
- *When [the character of] Retselitsoe had developed symptoms of illness, his elderly grandmother had to sling him on her back and walk for several hours to the nearest rural health clinic. [But] Retselitsoe was dead upon arrival… There is such a large gap in accessibility of healthcare for Basotho, as many of them must make very treacherous and lengthy journeys just to receive treatment…*
- *The book has helped me understand how we, Americans, take the little things for granted. For example, McGrath mentions how “bikes are a rare sight; not many people in town have the resources for small luxuries like these” (p. 155). This is not the case for people in America, especially in San Francisco. Our city has created bike lanes for our streets. Despite not having these small luxuries, Basotho people have found other ways to entertain themselves, like playing scrabble games and having a party. Chapters “Party Crashing, or The Kingdom of Lesotho,” “Midnight Basotho Dance Party,” and “Killing a Pig” all involve a party. These chapters show that Basotho people really value being surrounded by their community.*

Students described other cultural norms, including touching or holding hands with even casual acquaintances, slaughtering an animal for a celebration, the use of physical punishment in schools, how gender stereotypes can stigmatize individuals who do not conform (e.g., a man taking care of a child), expectations around funerals, and respect for elders. Having read the book early in the course allowed students to connect what they were reading to actual experiences, such as when strangers would greet students as they walked to class (even stopping to talk), seeing herd boys wearing traditional Lesotho blankets taking care of their goats, and participating in a send-off party at the end of the course.

***Session Review.*** Working in pairs, students presented key points from the previous day. They were instructed to include a photo illustrating anything they had seen or experienced. This exercise helped students to develop skills in discerning essential ideas, synthesizing and connecting them to create meaning and improve retention, and identifying “muddiest points” from the previous day’s activities or assignments. The review sessions prompted further discussion of topics like how LeBoHA sets priorities for intervention, the role of community health workers, and how HIV stigma and discrimination affect care-seeking.

This assignment encouraged active learning because it fell on the students to determine the key points rather than the instructor. For example, the session review of the health center trip provoked good discussion of barriers to access and retention in care, including gaps in staffing (with root causes involving a long and complicated recruitment process for nurses, inadequate number of posts, low wages, and geographic inequalities); transportation (causing difficulties getting sputum tests to the laboratory for diagnosis of TB); and patient living conditions and perspectives (food insecurity and concern that TB medications will darken skin tone). Having LeBoHA staff attending every session review facilitated an informed debate about individual, interpersonal, community, and policy-level factors affecting access to care.

***Teach Back.*** Each student presented findings and facilitated a discussion about an article on Lesotho’s health system or a particular health problem. Table 2 includes the topics and articles discussed. This assignment challenged students to summarize essential information related to a new topic, improving their own knowledge and understanding while also helping others to understand. To facilitate the Teach Back assignment, USFCA faculty gathered over 80 articles and reports on a range of topics related to local health issues, including demographic and health survey results, human resources management, health systems strengthening, HIV/AIDS and tuberculosis, other infectious diseases, noncommunicable diseases, food and nutrition, maternal and child health, primary care, sexual/reproductive health and interpersonal violence, quality improvement, and public–private partnerships. Students were welcome to choose one of the articles provided or to find an article on their own. Most students chose an article from the list provided. They needed to develop and give an informative presentation about the topic and engage other students, faculty, and staff in discussion. Students said they gained confidence by presenting, answering questions, and facilitating discussions during the Teach Back. One student designed a quiz that stimulated everyone to recall information. Sometimes the discussion brought up other, more recent research results, and identified areas where researchers need to probe more deeply, for example, the meaning of the correlation between solid fuel use and anemia in children [28, 29]. In the session on HIV/AIDS and the Lesbian, gay, bisexual, transgender (LGBT) community, LeBoHA instructors helped students appreciate the challenging task of confronting negative social judgments, and how best to engage people living with HIV/AIDS in this type of environment.

**Table 2:** Teach Back Topics and Articles.

TOPICS	ARTICLE
Sexual violence	Brown, L., Thurman, T., Bloem, J., & Kendall, C. (2006). Sexual violence in Lesotho. *Studies in Family Planning, 37*(4), 269–80.
Digital health interventions for sexual and reproductive health	Nkabane-Nkholongo, E., Mokgatle, M., Bickmore, T., et al. (2023). Adaptation of the Gabby conversational agent system to improve the sexual and reproductive health of young women in Lesotho. *Frontiers in Digital Health, 5*, 1224429.
Tuberculosis	Andom AT, Gilbert HN, Ndayizigiye M et al. (2023). Understanding barriers to tuberculosis diagnosis and treatment completion in a low-resource setting: A mixed-methods study in the Kingdom of Lesotho. *PLoS ONE, 18*(5), e0285774.
Child health and anemia	Letuka, T. & Frade, S. (2020). Household and individual risk factors of anaemia among under-5 children in Lesotho. *African Health Sciences, 20*(3), 1478–1486.
Health financing	World Bank. (2017). Lesotho Public Health Sector Expenditure Review. Washington, DC: World Bank.
Health statistics	Ministry of Health [Lesotho] and ICF International. (2016). 2014 Lesotho Demographic and Health Survey Key Findings. Rockville, MD: Ministry of Health and ICF International.
HIV/AIDS and the LGBT community	Logie CH, Perez-Brumer A, Mothopeng T, et al. (2020). Conceptualizing LGBT stigma and associated HIV vulnerabilities among LGBT persons in Lesotho. *AIDS and Behavior, 24*, 3462–3472.

***Reflection.*** Reflective writing is an analytical practice in which a student describes events, interactions, and thoughts, and adds personal thoughts on their meaning. Reflection offers students the opportunity to consider how their personal experiences and observations shape their thinking, to identify preconceived ideas and assumptions they may have had and to self-assess their emotional reactions and ability to cope [30]. This is central to Jesuit pedagogy and the recursive process of experience, reflection, and action [31]. It is also an important approach to global health learning assessment [32].

Students wrote two reflective essays on what they were experiencing and learning during the program. Guiding questions and structure were provided (Table 3). In the first essay, students were asked to consider each of their senses as they reflected on their experience in the program along with other questions. In the second essay, students reflected on their learning using Fink’s framework of significant learning [33].

**Table 3:** Guidance for Reflective Essays.

ESSAY AND TIMING	GUIDING FRAMEWORK AND QUESTIONS
Reflective Essay 1, due one week after arrival	**Framework: The Five Senses** Using each of your senses, **touch, sight, hearing, smell, and taste**, describe something you have experienced so far in Lesotho. Write about why this was salient or interesting to you. What has happened so far that was **surprising or unexpected**? Reflect on one experience and what you draw or conclude from it. Reflect on what you are learning about the **types of assistance needed for health systems strengthening** in Lesotho. What questions do you have, or topics do you want to learn more about to better understand the challenges the country faces and ways to surmount them?
Essay 2, due the last day of the program	**Framework: The Significant Learning Model** Reflect on what you are learning during the Lesotho Immersion, according to the six dimensions of Fink’s significant learning model. These include: **Foundational knowledge**: “Understand and remember” learning (such as facts, terms, and concepts) **Application**: Thinking (critical, creative, practical—problem-solving, decision-making) **Integration**: Making “connections” (i.e., finding similarities or interactions among subjects or concepts) **Human dimensions**: Learning about self (differences learning has made in intellectual, ethical, or professional development); understanding and interacting with others **Caring**: Identifying/changing one’s feelings, interests, values; high and low emotional moments **Learning how to learn**: Becoming a better student, learning to ask and answer questions, and becoming a self-directed learner How has the experience in Lesotho helped you to **grow professionally**? What aspects of the program have or will influence your work and career?

For the first essay, students made many observations about senses, recalling the beauty of the lush landscape and the sight of babies being carried on their mothers’ backs; the noise of severe thunderstorms and the clicks of the Sesotho language being spoken; the smell of crisp air and earthy soil after rain, and heavy smoke from wood-burning cooking fires; the feeling of a soft, traditional mohair scarf; the taste of “fat cakes” (fried dough balls), lamb, and traditional South African samp (dried corn kernels that have been pounded or stamped until broken, eaten with stew). Others mentioned seeing the peeling paint and missing equipment in the hospital, acknowledging how it saddened them but also helped them appreciate the challenging work environment. The initial essay served the intended purpose of helping students use all their senses to make meaning of what they were experiencing.

The quotes below provide other examples of insights gained through reflections:

- *It was difficult not to notice the differences between this hospital and hospitals back in the United States…. to see how things I take for granted in the United States when I am working at the hospital would be seen as a luxury here.*
- *When passing by the outdoor cooking stalls, I was surprised at how potent the smoke was and developed concern for the air quality.*
- *I began to wonder why the power kept going out. What is the system for power outages? I think about how much our health care systems rely on power to utilize medical equipment, electronic health records, and light to perform procedures.*
- *If you listen closely, you can hear cowbells in the distance. That sound tells me that where there is a cow, there is a shepherd, dressed in his traditional tribe blanket. When I hear this sound, I wonder how many people this shepherd is providing for and if he makes a decent wage.*

In the second essay, students reflected on their own learning using Fink’s framework (see Table 3).

They identified foundational knowledge topics, such as the difference between incidence and prevalence; how the lack of a national death registry affects mortality statistics; quality improvement tools; and the critical importance of government policies for human resources for health. One student reflected on learning about how difficult it was to get doctors to come back to Lesotho after training in South Africa, leading the government to hire foreign doctors: “I am curious if the reliance on foreign doctors creates challenges to provide culturally appropriate care. There could be language barriers. Perhaps people would be more trusting of a Basotho doctor and more likely to come in for an appointment or heed medical advice.” Other students mentioned statistics that stuck in their minds about health access (“only 1 in 10 Basotho have cars”) and patient perceptions (“One third of Basotho women and 40% of Basotho men think a husband can be justified in beating his wife.”).

For learning in the domain of “application,” one student mentioned preparing for the session review: “Coming up with a muddiest point for my session review required me to think critically and deeply about what I had learned the previous day and consider the feasibility of various ideas and seek any necessary clarifications.” Students mentioned preparing for the Teach Back and having to think not only about what to teach, but how to create a fun and engaging environment for learning by adding a quiz or discussion questions. Two students mentioned the final presentation. One said “My group members and I conducted a lot of research to look for additional solutions to the issue of transportation barriers that affect healthcare. I have done more research in this course than I have during my undergraduate program, so it has been a great experience for me to implement more evidence-based solutions into my schoolwork.”

In considering “integration” one student wondered how facility ownership affected access to contraceptive services, given that 38% of Lesotho’s hospitals are affiliated with the Christian Health Alliance of Lesotho. Several students mentioned how they assessed commonalities and differences between the US and Lesotho healthcare systems. One noticed similarities in TB care strategies: “I think about how [in California] we usually have health workers that can meet people at their homes to collect TB sputum and DOT [directly observe therapy]. It is similar in Lesotho in that the village health workers can help with sputum collection and DOT.”

On the human dimensions of learning, one student was enthusiastic about the international nature of the program. “Being in Lesotho to observe, walk around, consume the food, connect with Basotho people, and visit the health care facilities has helped me to understand more about Lesotho. In turn it has helped me to appreciate and respect the culture and its people.” Another student was struck by the collegial working environment at LeBoHA and reflected on her own privilege: “This experience has opened my eyes to how NGOs run and what challenges they face. I’ve learned that it is possible to find a workplace where staff enjoy interacting with one another and can be serious about changing healthcare delivery. I also understand my privilege and opportunities that I have living in the U.S., and I would like to use them to help others.” Some students mentioned adapting to local norms, such as greetings, as shown in the two quotes below.

*As a child, my New Yorker dad taught me to ignore people on the street. [But] the greetings here feel more like they come from a place of genuine curiosity. I have had to modify my mindset on interacting with others. Transitioning from ignoring people on the street to saying “hello” back has required me to question my gut reaction to ignore people on the street.*

*I love that strangers in Lesotho will greet anyone they see on the street or walk past. It’s so different compared to the individualistic culture in the United States. I have felt so honored that we have been met with kindness and generosity in the interactions we’ve had here.*

Privilege was also mentioned in reflections on the dimension of caring. One student observed:

*I’ve learned so many new things in Lesotho, many of which have made me have to stand back and reevaluate the privileges that I have been fortunate enough to have as an American, like the multitude of healthcare resources and small mortality rate present in the*

*healthcare system in the U.S. It’s been really disheartening learning about how children and women have died because of the constraints that are present in Lesotho healthcare. I have learned to be much more understanding and compassionate in my approach to healthcare and life as well.*

Other students noted that learning about Lesotho’s health situation, including high rates of mortality and the causes of disease, has evoked feelings of empathy, compassion, and a sense of responsibility. One student noted “Due to my family’s difficulties navigating the healthcare system here in the U.S., I naively thought I knew pretty much everything about public health. Plot twist: I did not. Coming to Lesotho allowed me to see firsthand the damage that a lack of a well-established healthcare system does to individuals. It has strengthened my interest in public health.”

Finally, considering the dimension of learning how to learn, several students mentioned that they became better learners by asking questions and participating in discussions. The quotes below illustrate how students found courage to ask questions and appreciated their value.

*Sometimes I can be shy about asking questions or bringing up controversial thoughts on challenging issues. I have learned that if done with the best intentions, a curious mind, and objectively, it can be helpful to speak up and engage rather than fear that it is a “stupid”* *comment or question. Being in Lesotho and observing a different culture, asking more questions helps me to avoid making assumptions.*

*I’ve improved as a student throughout this experience. I’ve learned the value of asking thought-provoking questions that can lead to a discussion. I also enjoyed learning about the healthcare services and viewing the clinics in person to see things firsthand.*

A nursing student mentioned learning to learn through the Teach Back: “I learn much more when I am able to test my knowledge by teaching it to others…. I am now much more open to learn in different places, different circumstances, and different subjects as well!”

The second essay allowed students to reflect on how the course affected their professional goals. One student wrote

“*I was unsure if I wanted to pursue a career in global health and this course helped solidify my interest. It gave me more insight into global health as a career and insight into the various aspects of global health that I could specialize in. I don’t think I could have gained this insight from just taking a course in the US. It was especially meaningful to do it abroad and be immersed in it.”*

***Final Presentation.*** In two groups, students tackled a health systems problem facing LeBoHA and the people of Lesotho. They provided background, conducted a problem analysis, analyzed workable solutions, and made recommendations. The LeBoHA faculty and staff suggested topics, and students were also welcome to choose their own. The two presentations were on “Harmony in health: Creating a robust disease and mortality registry,” and “A ride for all: Innovative transportation methods to overcome healthcare barriers in Lesotho.” Students searched the literature, applied teamwork skills, and used revision strategies to develop a concise and informative slide deck.

These assignments were spread throughout the course to engage students and encourage them to make connections as they were learning. This timing also allowed students to receive early feedback on performance expectations. Students taking the course for graduate credit and/or more units did extra work, including an extra Teach Back session and organizing a focus group to collect feedback on the course.

### Student ratings and feedback

Table 4 presents average student ratings from standard USFCA course evaluation forms completed at the end of the course. The students rated each statement on a Likert-like six-point scale, from 1 (=strongly disagree) to 6 (=strongly agree). These were translated into a percentage score.

**Table 4:** Student Evaluation Data.

DIMENSIONS OF LEARNING	AVERAGE SCORE OUT OF 6 (%)
**Instructional Design**	
Learning outcomes were clearly stated.	5.71 (95%)
Student responsibilities were clearly defined.	5.57 (93%)
The course schedule was clearly laid out.	5.00 (83%)
Criteria for assessing performance were clearly stated.	5.71 (95%)
**Comments** The syllabus clearly stated the learning outcomes. The assignments had clear instructions about what our responsibilities were. Grading rubrics were included in assignments. There was a day-to-day schedule that was followed clearly. There was variability …but it was not a deterrent to our education. The course schedule was laid out when we arrived, but there were certain changes at the last minute.	
**Instructional Practices**	
The course’s subject matter was covered in a clear manner.	5.14 (86%)
Course sessions were well prepared.	5.57 (93%)
Feedback on this course was constructive.	5.43 (90%)
**Comments** The content was covered clearly. It was all well prepared. All lectures were presented in a timely manner and allowed time for discussion. Feedback was very timely. There was a slight disconnect between some articles provided for the Teach Backs and the opinions/feedback given by some LeBoHA staff members.	
**Student Engagement**	
Instructional activities contributed to my desire to engage in the course.	5.29 (88%)
This course stimulated my interest in the subject matter.	5.71 (95%)
This course motivated me to learn.	5.71 (95%)
**Comments** The activities we did were interesting. I learned so much about global health. [The course] piqued my interest. Allowing me to immerse myself in the clinic setting and view the way the healthcare system worked was great! It added to more interest in the subject and understanding of the content being presented as well. It motivated me to learn more about global health and health administration. There was a little too much lecture for my liking. I would have liked more interactive elements.	
**Student Learning**	
I increased my knowledge of this subject as indicated by the course learning outcomes.	5.86 (98%)
Strategies for learning (learning how to learn) on this course are transferable to other subjects.	5.86 (98%)
This course contributed to my understanding of the subject matter.	5.86 (98%)
**Comments** We did a lot of reflections, which is how I like to learn. Learning and improving the skills and techniques of teaching and note-taking allowed for further discussion and understanding of content and materials. This class was a great experience to view the hospitals and the way the health systems work as well as the lectures about the health-care sector. [We learned] through viewing the changes that need to be made and how to impact the systems and develop further improvements. It also allowed us to view the setbacks in health care and how the community is going about to change the system. This class was amazing. I learned so much about the culture of Lesotho and the public health system in Lesotho. I enjoyed this course and traveling to Lesotho! It has positively made an impact on my professional and personal goals.	

Overall, students rated the program highly. Comments indicated that students liked having a predictable schedule and wanted more interactive engagement. A focus group held by the students to gather more informal feedback suggested that future programs include additional opportunities for experiential learning and expand student preparation for the experience, possibly through Sesotho language instruction and interaction between students and LeBoHA faculty and staff prior to arrival.

The “disconnect” mentioned by one student refers to the fact that Teach Back presentations could spur debate. During the Teach Back, Lesotho staff sometimes provided more recent data and questioned the assumptions from the articles. Since students had chosen articles from a prepared list, they were concerned about being shamed or judged negatively when in fact they were following directions [34]. In the future, the assignment directions should explain that debate is desirable, and disagreement is a part of healthy dialogue. The assignment might even prompt students to ask staff if they have more current data or insights to add. This framing could help make students feel more confident in facilitating without fear of being wrong or making a mistake.

## Discussion

This article described the design and implementation of a short-term global health immersion in Lesotho on health systems strengthening. The program aimed to have a partnership that provided (1) mutual benefit between the two institutions, (2) relevant curriculum and experiential learning about health systems challenges in Lesotho, and (3) opportunities for interprofessional collaboration between undergraduate nursing and graduate public health students to promote cultural awareness and humility and foster global perspectives and relationships.

### Mutual benefit

We pursued the goal of mutual benefit through close collaboration during the design process. For example, seeking input and advice from LeBoHA early in the process allowed USFCA to shift the focus from quality improvement to health systems strengthening. This avoided pressure on local health professionals’ time [35] and permitted more flexibility in scheduling. LeBoHA staff chose the extracurricular activities and field visits, to capitalize on their knowledge of the locality and opportunities for engaged learning.

The program allowed LeBoHA an opportunity to evaluate short-term immersion courses as a possible new revenue stream aligned with LeBoHA’s educational mission. LeBoHA staff time was not explicitly budgeted as a line item. This might be considered more carefully in future budget development [27].

It is important to ensure financial fairness between organizations in shouldering the costs of the program. At the same time, the program fee and travel costs already deterred some students from participating. USFCA and LeBoHA should consider other sources of revenue to build enrollment and ensure the local partner is adequately compensated. One strategy could be to provide an academic appointment to a Basotho faculty member to replace the USFCA faculty [35]. The fact that this was a credit-bearing program, as opposed to fee-only, opens the possibility of tuition revenue sharing to benefit the local institution. The program might also seek philanthropic support for student travel grants, as did a long-running program for clinical immersions in Kenya [4].

Another benefit of the program for LeBoHA faculty and staff is the opportunity to develop skills in designing the curriculum for the immersion program and to hone teaching skills as they prepare lectures on health systems strengthening topics. In future programs, peer review of teaching could be incorporated, including both USFCA and LeBoHA faculty, with a focus on curriculum design, inclusion of active-learning exercises, and giving feedback to students.

Articles about global health immersions often focus more on the student experience and do not specifically address mutual benefit; however, an article about a global health immersion in Belize described mutual benefit to three sets of stakeholders: “indigenous populations’ health-related needs were addressed; nursing faculty experienced personal and professional growth; and students achieved service-learning outcomes and developed skills necessary for entry-level public health nursing practice [9].”

All global immersion programs should try to assess mutual benefit. For example, it may be helpful to solicit voluntary, anonymous feedback from those involved in the design and implementation of the program [2]. Experts recommend using a process, even with small partnerships, to monitor and evaluate the nature of the relationship and identify possible power imbalances and inequities [36]. Addressing identified barriers can help smooth the path for shared decision-making, ownership, organizational learning, and capacity development.

### Relevant curriculum and experiential learning

Considering program relevance, students viewed the objectives and content of the global health immersion as relevant and said that the design of the course helped them gain foundational knowledge, apply and integrate concepts through assignments that tapped into problem-solving and creative thinking, and engage outside the classroom through field visits and other events. The final presentation assignment gave students an opportunity for experiential learning—the process of acquiring skills, knowledge, and experience through firsthand engagement within a competency or vocation—as they worked on possible solutions to health systems challenges in Lesotho [37].

Like other immersions in Ethiopia and Tanzania, students appreciated activities such as walking to class each morning and shopping in local markets. These were opportunities to learn about the culture and people of Lesotho [6, 7].

One aspect of learning that was not fully realized was the incorporation of a photo into the session review. The purpose of this exercise was to practice visual thinking strategies, a practice that can be especially helpful to launch conversations about diversity and social determinants of health [38, 39]. Students leading the review were asked to incorporate a photo so that other students could observe, discuss, and offer alternative interpretations. However, most of the time students simply used photos to illustrate the review, e.g., a photo of students presenting a Teach Back from the previous day. They did not use the photo as a learning experience. To incorporate visual thinking more effectively, future immersions should provide more instruction for students on the goal and application of visual thinking as a professional skill, i.e., add specific questions that students should ask themselves about the photo, such as “what’s going on in this photo?” and “what makes you say that?,” then revealing the true “backstory” of the photo [40]. We should also help students conducting the review on how to plan the time needed for students to observe, discuss, and make meaning from the photo being shared.

Patience, flexibility, and resilience are important qualities for success in global health [41]. Although students were warned that flexibility might be needed in Lesotho, some were still unsettled when the schedule changed (for example, not knowing the exact departure time for a trip). While interruptions, delays, and changes in plans are normal in global health, in the context of a short educational program, students may feel the lost opportunity more deeply. Faculty may need to discuss and process emergent schedule changes with students, to help them see the changes as a learning experience rather than a loss. An immersion program in Belize also noted the importance of flexibility and adaptability on the part of both students and faculty [9]. This may mean more advance planning for local approvals if students are to be engaged in clinical work.

### Interprofessionalism, cultural awareness, and global perspectives

We did not use evaluation tools specific to the goals of promoting competencies in interprofessionalism and cultural awareness. Instead, we applied a standard tool used by USFCA for evaluating all courses. This tool, while gathering perceptions of students on course design, pedagogy, engagement, and student learning, did not include questions specific to the learning dimensions of interprofessionalism, cultural awareness, or specific global health perspectives.

Future programs should be designed to include a pre- and post-test of knowledge, attitudes, and practices of students in these areas. For example, in their study of a global immersion in Nicaragua, Leathers et al. (2018) implemented pre- and postsurveys aligned with Interprofessional Education (IPE) competencies [10, 42]. The Nicaragua study found statistically significant increases in several IPE competencies, including “Effectively communicate your own profession’s roles and scope of practice to other health professionals,” and “Engage other health professionals in shared patient-centered problem-solving [10].” That program was designed for medical and nursing students, but questions could be adapted for nursing and public health professions.

For cultural awareness, while student assignments provided qualitative evidence of gains in cultural awareness, the program could assess further using pre- and post-test questions adapted from the Cross-Cultural Competence of Healthcare Professionals survey instrument [43] or other similar tools [44–46]. A two-week interdisciplinary immersion in Tanzania used this assessment method for their service-learning program, involving nursing, dental, and physician assistant students [8]. Researchers documented improvements in students’ ability to describe their role as global citizens and demonstrated better understanding of complex global health issues, local history, and culture [8].

In professional settings, international experiences have also been known to develop global perspectives and skills, such as a widened horizon, self-confidence, flexibility, and tolerance [47]. An evaluation of the Pfizer Global Health Fellows international volunteering program found that the program helped employees develop confidence and ability to operate in conditions of uncertainty and to perform even when faced with poor logistics, inadequately defined assignments, and other constraints [48]. These might be additional areas to assess through pre- and post-testing for program impact.

This immersion program is also linked to requirements of national accrediting bodies for public health and nursing, the Council on Education for Public Health (CEPH) and American Association of Colleges of Nursing (AACN), respectively. Both CEPH and AACN require competencies or domains in interprofessionalism. CEPH requires that MPH students be able to “integrate perspectives from other sectors and/or professions to promote and advance population health [49].” The AACN describes interprofessionalism in nursing as “collaboration across professions and with care team members, patients, families, communities, and other stakeholders to optimize care, enhance the healthcare experience, and strengthen outcomes [50].”

In their final presentations, students practiced additional CEPH competencies applying awareness of cultural values and practices to the design or critique of public health programs, practicing cultural humility in communicating public health content, and communicating audience-appropriate public health content to a nonacademic audience [49]. The AACN also emphasizes systems-based practice in nursing to effectively coordinate resources to deliver quality care to diverse populations, which is aligned with the health systems strengthening focus of the immersion program [50]. Future offerings of the program can assess these accreditation requirements for public health and nursing to demonstrate direct relevance to national curricular standards.

### Assets, pitfalls, and challenges

We had several assets in developing the Lesotho immersion program, including the strong support of the Vice Provost of Global Education, Immersions and Strategic Initiatives and the Dean of the School of Nursing and Health Professions at USFCA. We also were able to tap into the existing infrastructure for the operation of global immersion programs at USFCA, including a website for enrollment and communication with students, contracting and legal review, travel insurance, and orientation resources. Additionally, we were able to use preexisting course numbers for undergraduate and graduate enrollment in a global health immersion, saving time on curriculum review and administrative steps. Finally, the facilities at LeBoHA (classrooms, meal preparation, and local transport) were excellent, and the connection of the program director with LeBoHA staff facilitated trust between the local partner and the University.

Challenges arose due to the opacity of USFCA financial management systems. The USFCA School of Nursing and Health Professions could not charge expenditures directly to the Center for Global Education, and some unexpected expenditures incurred by the Center for Global Education appeared on the books of the School of Nursing and Health Professions. The distribution of costs should have been made clear between departments from the beginning. Recruitment was challenging due to the cost of the program fee, transportation, and tuition. At USFCA, undergraduate tuition during intersession is higher than during regular semesters for unknown reasons. This type of policy can be a barrier to access for students.

In the country, challenges were few. We considered local holiday schedule when planning the program, yet still some disruptions and changes occurred when some students missed a connecting flight to Lesotho, and when LeBoHA staff needed to travel to defend their budget at the Ministry of Health during the program. Heavy rains affected Internet access. These disruptions/changes were framed as normal challenges to working in global health and a lesson in adapting a flexible mindset.

## Conclusion

The experience of the Lesotho Global Health Immersion shows that short-term global health immersions can help to prepare US nursing and public health students for careers in global health or working with underserved communities in the United States. Immersion experiences must be developed in partnership and provide reciprocal benefits to the institutions involved. Special attention should be paid to documenting student progress in developing cultural competency, interprofessional skills, and global perspectives.

## Additional File

The additional file for this article are as follows:

Supplementary File. Detailed Assignments.
