# Supplementary material for: A Global Health Immersion Program for Nursing and Public Health Students
Source: Ann Glob Health. 2025 Jul 18;91(1):37. doi: 10.5334/aogh.4746 (PMC12292055; doi:10.5334/aogh.4746)
Supplement: Supplementary File. — Detailed Assignments. [file agh-91-1-4746-s1.pdf]

Supplementary File: Detailed Assignments

A. Assignment 1: Reflections on Everything Lost is Found Again

Directions: Read the book "Everything Lost is Found Again: Four Seasons in Lesotho" (Will McGrath, Dzanc Books, 2018). Answer the study. There is no page limit, but I estimate it should take about 3 pages, double-spaced, to respond. Save your response in a Word file and upload to Canvas.

Study Questions

1. Consider the two quotes at the beginning of the book.

    > "Strange things happen to them, some bitterly cruel and some so beautiful that the faith is refired forever." (John Steinbeck, *The Grapes of Wrath*)
    >
    > "Violet learned then what she had forgotten until this moment: that laughter is serious. More complicated, more serious than tears." (Toni Morrison, *Jazz*)

    a. Choose **ONE** of the quotes and find examples from the book that illustrate. Discuss.
2. How did the book help you understand the poverty, hardships, and inequalities experienced by the people in Lesotho? What does the author perceive to be the strengths or assets of the country and its people? Explain and give examples.
3. Describe three Basotho values, rituals, or social/cultural norms illustrated in the book. How are they similar or different to values or social norms in the U.S.?
4. Several stories touch on individuals and families affected by HIV/AIDS. What are some of the challenges that HIV/AIDS has created in Lesotho?
5. Why did the author compare Tseli's kindergarten graduation to Easter? ("The Unlikely Graduation of Tseli Moeletsi")
6. What misconceptions did the author reveal in recounting the story of the scrabble game? ("Jink")
7. Pick one story or chapter from the book that surprised, puzzled, or made you uneasy. Briefly summarize the story and explain why it sparked this response.

Criteria for Grading

1. Complete, thoughtful, and relevant responses to each question, showing that you have read the book.
2. Support claims with examples or quotes from the text (citing page number).
3. Writing is clear, coherent, and concise, with no errors in grammar, spelling or syntax, and no typos.

B. Assignment 2: Journal Entries

Directions: Reflective writing is an analytical practice in which a student describes events, interactions, and thoughts, and adds a personal reflection on their meaning. Reflection offers you the opportunity to consider how your personal experiences and observations shape your thinking, and possibly to identify preconceived ideas and assumptions.

You will be asked to complete two reflections. Each reflection should be 1-2 pages for undergraduates, and 2-3 pages for graduate students. Answer the guiding questions.

Guide Questions for Journal Entry One:

1. Using each of your senses: touch, sight, hearing, smell, taste, describe something you have experienced so far in Lesotho. Write about why this was salient or interesting to you. Use sub-headings (e.g. touch, sight, etc.) for each observation.
2. What has happened so far that was surprising or unexpected? Reflect on one experience and what you draw or conclude from it.
3. This question is required for MPH students (optional for undergraduates). Reflect on what you are learning about the types of assistance needed for health systems strengthening in Lesotho. What questions do you have, or topics do you want to learn more about to better understand the challenges the country faces and possible ways to surmount them?
4. Add any other observations, thoughts or reflections you would like to share.

Guide Questions for Journal Entry Two:

1. Read the two-page summary of significant learning.
2. Reflect on what you are learning during the Lesotho Immersion, according to the six dimensions of Fink's significant learning model (foundational knowledge, application, integration, human dimensions, caring, learning how to learn).
3. This question is required for MPH students (optional for undergraduates): How has the experience in Lesotho helped you to grow professionally? What aspects of the program have or will influence your work and career?
4. Add any other observations, thoughts or reflections you would like to share.

Grading criteria:

1. Assignment shows thoughtful personal reflection.
2. Assignment follows directions and answers guide questions.
3. Journal entry has no errors in grammar, spelling or syntax, and no typos.

C. Assignment 3: Session Review

Directions: Each morning, after the first day, a pair of students will review the previous day. The summary should take about 10 minutes, and will include the following:

1. Key points from the previous day
2. A photo or photos of interest from the previous day, with explanation or caption(s)
3. A "muddiest point" that needs to be clarified, any questions from previous day, or a reflective note on culture, weather, social life, etc.

Think about 3Ts, Triage, Tools, and Tell Me, when planning the session review.

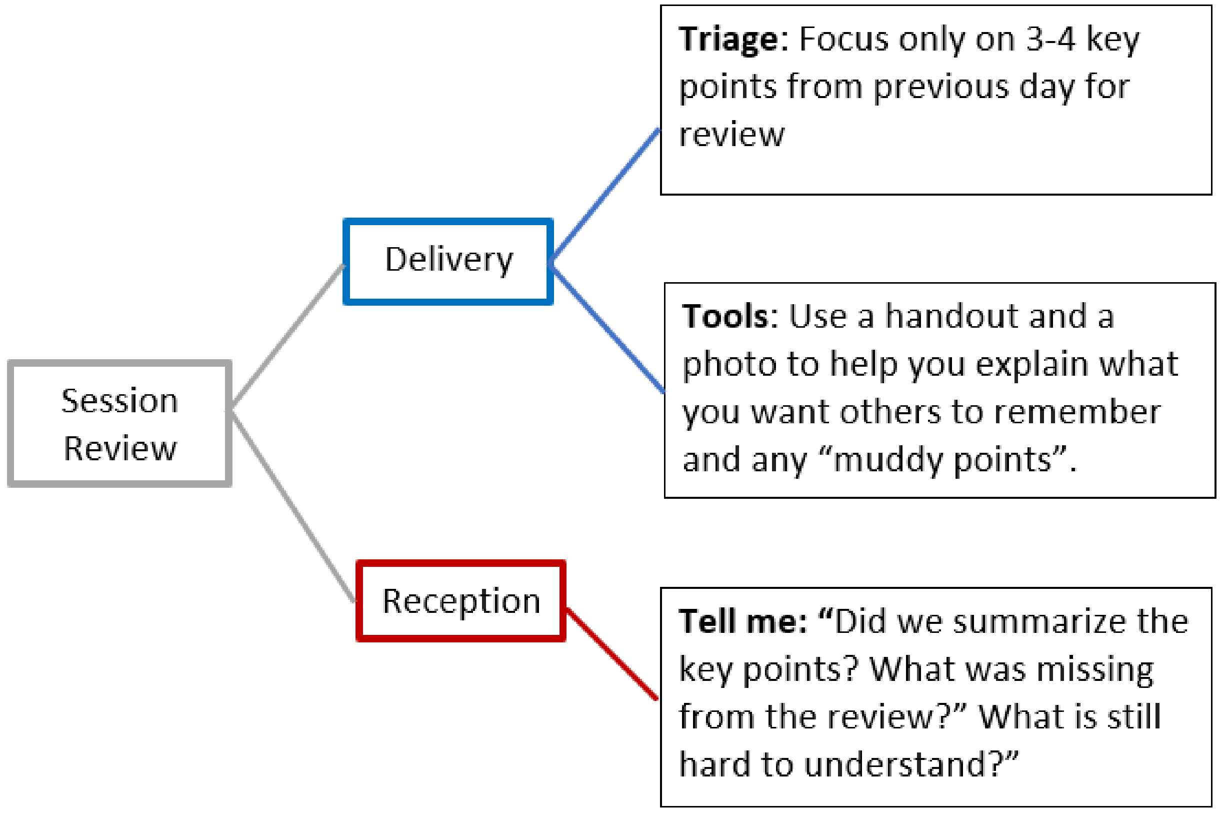

The key points summary or handout should be uploaded to Canvas on the day the pair is presenting, including the photo(s). In class, the pair presenting can share the file with others and summary can be done verbally with or without visual aids or handouts, depending on technology available.

Criteria for grading

1. complete, succinct summary of key points,
2. relevant photos with explanation/caption,
3. thoughtful question or reflective note that pertains to session content and experiences.

4. neatly formatted, names on the inside of document, no typos or grammatical errors, file name includes both students' last names and the name of assignment.

D. Assignment 4: Teach Back

This individual assignment requires students to choose one reading, summarize key points (since others may not have read the reading), and lead a discussion on the points raised/answer questions. The Teach Back should take about 10 minutes , plus about 5 minutes for questions or discussion. One or two students will be assigned to do the Teach Back for each session. See list of possible articles on Canvas, or feel free to choose your own.

Directions:

1. Skim the list of readings to find an article that is interesting to you. All articles are available on Canvas in the Readings folder and sub-folders. You may need to read several articles to find one that you understand well and think you would be able to summarize and discuss.
2. Write notes to summarize the article you have read and to help you present orally. Focus on these four points:
   - Topic (what is topic of the article--tell students the title and authors)
   - Objective and Methods (What were the objectives or purpose of the study? What were the methods used to conduct the study? Was the study quantitative (a survey?) or qualitative (interviews, focus groups?) When were data collected? Tell us about the population studied, i.e. age, gender, residence, other attributes. Please let me know if you need help in understanding methods and how to summarize them.
   - Findings (pick the most important, interesting, or surprising findings, and describe them. Use data to support the findings, if the study provides this, e.g. statistics, quotes)
   - Questions or discussion points (Tell us why you find this important, interesting, or surprising? Is the article controversial? Why? What are the implications for health care or health systems strengthening in Lesotho? How does this relate to what you have already learned in program? Prepare a question or two to promote discussion. Ask students if they have questions for you.)

Criteria for Grading: The presentation of the Teach Back will be graded based on:

1. Accurate, succinct summary of methods and findings, with appropriate supporting data
2. Quality of oral presentation, including making eye contact, minimal use of notes, strong voice
3. Ability to ask and answer questions and promote discussion of key findings and why they are important, interesting, or surprising.
